# Deciphering the role of miR-71 in *Echinococcus multilocularis* early development *in vitro*

**DOI:** 10.1371/journal.pntd.0007932

**Published:** 2019-12-27

**Authors:** Matías Gastón Pérez, Markus Spiliotis, Natalia Rego, Natalia Macchiaroli, Laura Kamenetzky, Nancy Holroyd, Marcela Alejandra Cucher, Klaus Brehm, Mara Cecilia Rosenzvit

**Affiliations:** 1 Laboratorio Biología Molecular de Hidatidosis, Instituto de Microbiología y Parasitología Médica, Universidad de Buenos Aires-Consejo Nacional de Investigaciones Científicas y Tecnológicas (IMPaM, UBA-CONICET), Buenos Aires, Argentina; 2 University of Würzburg, Institute for Hygiene and Microbiology, Consultant Laboratory for Echinococcosis, Würzburg, Germany; 3 Institut Pasteur de Montevideo, Unidad de Bioinformática, Montevideo, Uruguay; 4 Wellcome Trust Sanger Institute, Wellcome Trust Genome Campus, Hinxton, Cambridge, United Kingdom; University of Cambridge, UNITED KINGDOM

## Abstract

Echinococcosis represents a major public health problem worldwide and is considered a neglected disease by the World Health Organization. The etiological agents are *Echinococcus* tapeworms, which display elaborate developmental traits that imply a complex control of gene expression. MicroRNAs (miRNAs), a class of small regulatory RNAs, are involved in the regulation of many biological processes such as development and metabolism. They act through the repression of messenger RNAs (mRNAs) usually by binding to the 3’ untranslated region (3’UTR). Previously, we described the miRNome of several *Echinococcus* species and found that miRNAs are highly expressed in all life cycle stages, suggesting an important role in gene expression regulation. However, studying the role of miRNAs in helminth biology remains a challenge. To develop methodology for functional analysis of miRNAs in tapeworms, we performed miRNA knockdown experiments in primary cell cultures of *Echinococcus multilocularis*, which mimic the development of metacestode vesicles from parasite stem cells *in vitro*. First, we analysed the miRNA repertoire of *E*. *multilocularis* primary cells by small RNA-seq and found that miR-71, a bilaterian miRNA absent in vertebrate hosts, is one of the top five most expressed miRNAs. Using genomic information and bioinformatic algorithms for miRNA binding prediction, we found a high number of potential miR-71 targets in *E*. *multilocularis*. Inhibition of miRNAs can be achieved by transfection of antisense oligonucleotides (anti-miRs) that block miRNA function. To this end, we evaluated a variety of chemically modified anti-miRs for miR-71 knockdown. Electroporation of primary cells with 2’-O-methyl modified anti-miR-71 led to significantly reduced miR-71 levels. Transcriptomic analyses showed that several predicted miR-71 targets were up-regulated in anti-miR-treated primary cells, including genes potentially involved in parasite development, host parasite interaction, and several genes of as yet unknown function. Notably, miR-71-silenced primary cell cultures showed a strikingly different phenotype from control cells and did not develop into fully mature metacestodes. These findings indicate an important function of miR-71 in *Echinococcus* development and provide, for the first time, methodology to functionally study miRNAs in a tapeworm.

## Author summary

Echinococcosis, caused by the larval stages of tapeworms of the genus *Echinococcus*, is a neglected disease that affects millions of people world-wide. These parasites show elaborate developmental features that rely on a complex control of gene expression. microRNAs are small molecules which have been discovered in the last decades and control gene expression in animals, plants and viruses. microRNAs are highly expressed in several tapeworms but their biological function in these parasites is unknown. Assuming that microRNAs will be important for parasite development, we analysed the function of these molecules in *Echinococcus multilocularis*, employing an *in vitro* model that mimics the first developmental transitions which occur in the human host. By artificially decreasing the concentration of the highest expressed microRNA, we observed phenotypic alterations and inhibition of development. In addition, we identified possible mRNA molecules targeted by microRNAs and found that some of these are known for being involved in developmental processes in other organisms. This work provides novel methodology to study microRNA function in tapeworms. Furthermore, highly expressed parasite microRNAs that are absent in the host but fulfil an important role in parasite developmental processes can serve as selective drug targets against the underlying diseases.

## Introduction

Echinococcosis represents a major public health and economic issue in many countries and is considered a neglected tropical disease by the World Health Organization (WHO) [1,2]. This zoonotic disease is caused by cestodes of the genus *Echinococcus*. The two major forms of the disease in humans are cystic and alveolar echinococcosis caused by *E*. *granulosus sensu lato* (s. l.) and *E*. *multilocularis*, respectively. To date, the primary focus for echinococcosis treatment relies on surgery of the metacestode or chemotherapy using benzimidazoles [3]. Alveolar echinococcosis is a life-threatening zoonosis prevalent in the Northern Hemisphere where foxes act as definitive hosts [4]. Infection of the mammalian intermediate host (rodents and humans) is initiated by oral uptake of infectious eggs, which contain the oncosphere larva. Upon hatching from the egg in the host intestine, the oncosphere penetrates the intestinal epithelium and gains access to the host organs. Typically within the liver the parasite then undergoes a developmental transition towards the metacestode stage which is entirely driven by parasite stem cells (germinative cells) that have been carried to the host by the oncosphere [5]. As an asexual multiplication stage the metacestode grows multivesicularly and infiltrates the surrounding host tissue, eventually leading to organ failure. In natural rodent infections, head regions of the future adult worm (protoscoleces) are formed from the cellular germinal layer of the metacestode, and are subsequently taken up when the definitive host takes its prey [6]. A crucial role of germinative cells in parasite development and metacestode formation has previously been demonstrated. In particular, the germinative cells were shown to be the only mitotic cell type of *Echinococcus* which gives rise to all differentiated cells [5,7]. The *E*. *multilocularis* primary cell cultivation system [8], which initially contains around 80% germinative cells [5], is routinely used in developmental and immunological studies to mimic, *in vitro*, the transition of oncosphere-derived stem cells into metacestode vesicles [9–12]. Furthermore, due to their decisive role in parasite proliferation, the germinative cells are one of the most important cell types for the development of novel chemotherapeutics against echinococcosis [13]. The particular characteristics of the indirect and complex *Echinococcus* life cycle suggest that all these development processes require tightly controlled regulation of gene expression. MicroRNAs (miRNAs), a class of small regulatory RNAs, are involved in the regulation of many biological processes, primarily through the repression of messenger RNAs (mRNAs) by typically binding to the 3’ untranslated region (3’UTR) of target genes [14]. Upon binding of miRNAs to the 3’UTR, target mRNAs are either cleaved or, more common in metazoans, destabilized or translationally repressed [14]. The main determinant of this interaction is the seed region, an evolutionarily conserved sequence located in the 5′ end of the miRNA (nucleotides 2–8). miRNAs have already been identified in several cestode species including *E*. *multilocularis* [15] and *E*. *granulosus* s. l. [15–17]. In previous work, we found that miRNAs are highly expressed in all life cycle stages analysed, suggesting an important function in gene regulation of these zoonotic parasites. In addition, we found miRNAs differentially expressed between life cycle stages and species, and identified both parasite-specific miRNAs as well as several which are divergent from miRNAs of the host. We also previously predicted miRNA targets in *E*. *granulosus*, *E*. *multilocularis* and *Taenia solium*, using computational tools [18,19]. The studies on miRNAs in cestodes so far mainly focused on miRNA identification and computational predictions of target genes which inevitably leads to high false positive rates [20]. For this reason, there is still a lack of comprehensive functional and experimental validation of these putative miRNA targets. miR-71-5p (miR-71 from now on), a bilaterian miRNA absent in vertebrate hosts, is one of the top five most expressed miRNAs in the *Echinococcus* germinative layer and protoscoleces [15–17], suggesting that miR-71 may play stage-independent roles relevant to parasite homeostasis [15]. In addition, it was the miRNA with the highest number of predicted targets, suggesting that miR-71 controls several cellular processes in *Echinococcus* [18]. Studying the role of miRNAs in parasite biology and host interaction still remains a challenge. To this end, bioinformatic strategies adapted to each phylum as well as experimental strategies such as miRNA knockdown have to be developed [21]. Up to now, successful knockdown of platyhelminth parasite miRNAs has only been demonstrated for *Schistosoma japonicum* [22].

In the present study, we aimed to determine the role of miR-71 in *Echinococcus* metacestode development. To this end, we performed miR-71 knockdown experiments in *E*. *multilocularis* primary cell cultures followed by analysis of metacestode ontogenesis at the morphological level. In addition, using whole transcriptome and bioinformatic analyses, we identified potential target genes of miR-71 in *E*. *multilocularis*.

## Methods

### Ethics statement

All experiments in animals were carried out in accordance with European and German regulations on the protection of animals (Tierschutzgesetz, Section 6). Ethical approval of the study was obtained by the local ethics committee of the government of Lower Franconia (permit no. 55.2–2531.01-61/13).

### Animals and parasites

All experiments were performed with the *E*. *multilocularis* isolate Ingrid which was propagated in Mongolian jirds (*Meriones unguiculatus*) as previously described [8]. For miRNA identification and expression profiling of *E*. *multilocularis* primary cell culture isolate H95 was used. The isolation of parasite larvae from infected jirds was performed as previously described [7].

### *Echinococcus multilocularis* primary cell culture

Primary cell culture was performed as described in the previous publication [8], with some modifications. Briefly, metacestode vesicles in co-culture with RH-cells (rat hepatoma cells) were sieved with a tea strainer, washed twice with sterile PBS and incubated twice for 30 seconds in sterile distilled water. Then, metacestode vesicles were disrupted with a 10 ml pipette and centrifuged for 10 min at 2,000g at room temperature. Vesicles were washed again with PBS and centrifuged for 5 min at 250 g.

In order to achieve more standardized samples, the yield of the primary cell isolation process was quantified by measuring the cell density photometrically [8]. Using polystyrene cuvettes of 10 x 4 mm, (Sarstedt, Nümbrecht, Germany) 12.5 μl of the initial cell suspension were diluted with 987.5 μl 1xPBS. The optical density of this dilution was measured at a wavelength of 600 nm employing a Hitachi U-2000 spectrophotometer (New York, USA). One cell unit was defined as the cell suspension with a DO_600_ of 0.01. The *in vitro* cultivation of primary cells was carried out in 6-well plates (Nunc, Roskilde, DK) using 1000 cell units as starting material and 5.0 ml of cDMEM-A6/B4, a conditioned Dulbecco's Modified Eagle Medium used for fast primary cell growth as previously described [8]. The primary cells were placed in a closed Ziploc bag gassed with N_2_ and incubated at 37ºC overnight.

### Small RNA isolation, small RNA library construction and sequencing

**Fig 1:**
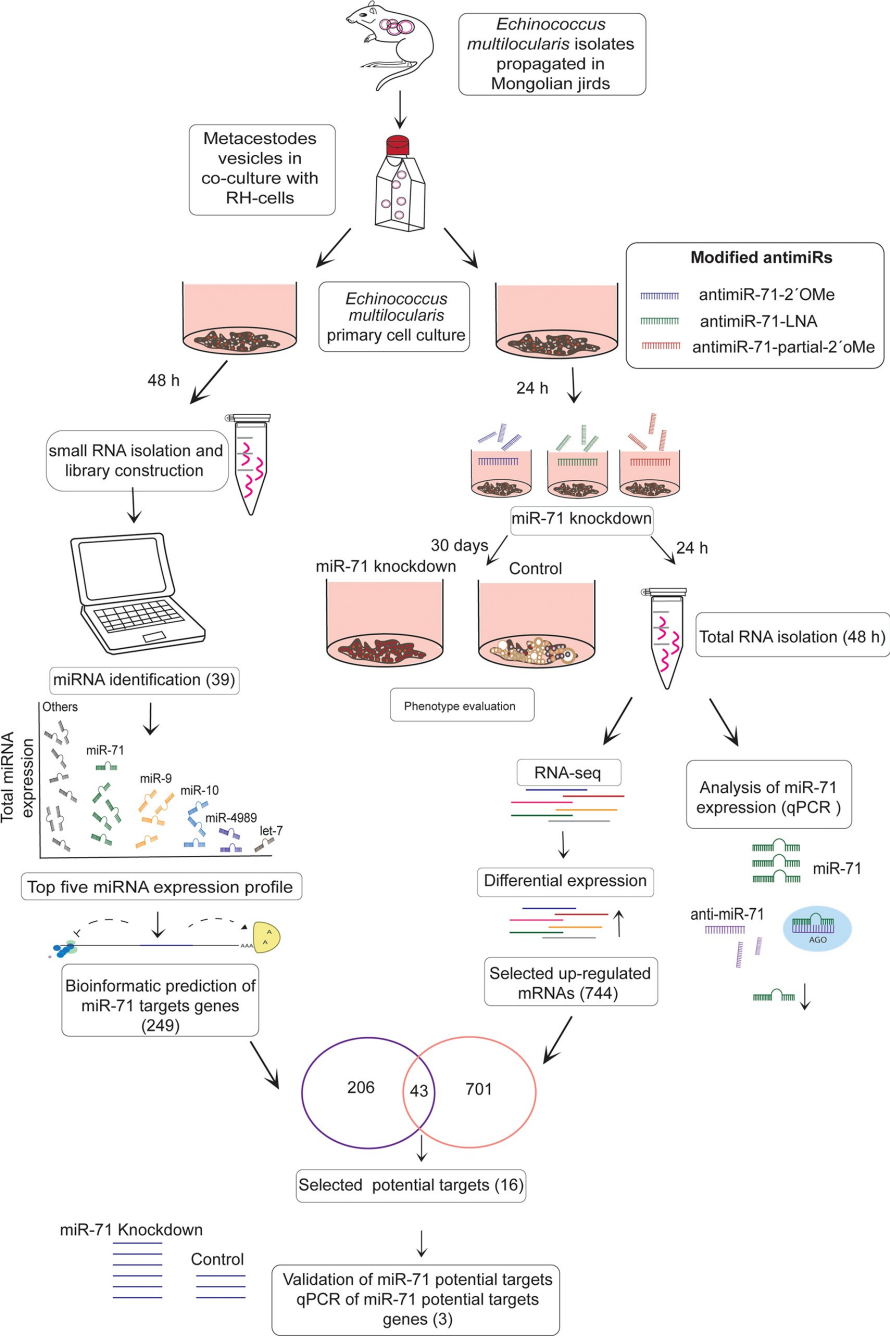
Workflow for identification of miR-71 potential target genes in *Echinococcus multilocularis* primary cell culture. The oligonucleotides complementary to miR-71 used were: anti-miR-71-2´OMe: 2’-O-methyl oligonucleotides in the complete sequence, anti-miR-71-LNA: locked nucleic acid and anti-miR-71-partial-2´OMe: 2´-O-methyl in some nucleotides. Three biological replicates were used for each experiment.

Small RNA isolation, library construction and sequencing of three biological replicates from 48 h primary cell cultures of *E*. *multilocularis* (Fig 1) were carried out as described in Macchiaroli et al. [23]. Briefly, cells were mechanically homogenized in Trizol (Invitrogen) for 10 s. Then, 200 μl of chloroform:isoamyl alcohol (24:1) were added and mixed thoroughly. Phase separation was carried out by centrifugation at maximum speed at 4°C. Then, 0.5× isopropanol and 4 μl of glycogen (5 mg/ml) were added to the aqueous phase and the RNA was pelleted by centrifugation at maximum speed at 4°C for 30 min. The resulting pellet was washed with 70% ethanol, air dried, and resuspended in nuclease-free water. The amount and integrity of total RNA was determined using a 2100 BioAnalyzer (Agilent, USA). RNA was concentrated by ethanol precipitation at -20°C overnight after elimination of polyadenylated mRNA using oligo-dT dynabead. Small RNA libraries were prepared using the NEBNext Multiplex Small RNA Library Prep Set for Illumina. Libraries were paired-end sequenced using an Illumina sequencing platform (HiSeq 2500) for 100 cycles.

### miRNA identification and expression profiling of *Echinococcus multilocularis* primary cell culture

In order to identify miRNAs from the small RNA libraries, the miRDeep2 software package [24] was used. Unique sequences were mapped to the *E*. *multilocularis* genome and used as input for miRNA prediction as previously described [15,16]. The initial miRDeep2 output list of candidate miRNA precursors of each library were manually curated to generate a final high confidence set of miRNAs retaining only candidate precursors with i) miRDeep2 score ≥ 4 ii) mature reads in more than one biological sample iii) star reads and/or seed conservation iv) no match to rRNA, tRNA or mRNA. The secondary structures and the minimum free energy of putative precursors and clusters were predicted using the mfold web server [25] and RNAfold software [26], respectively. To analyze miRNA expression, read counts of each individual miRNA in a sample (biological replicate) were normalized to the total number of mature miRNA read counts in that sample as previously described [16]. Then, normalized miRNA reads counts were averaged between the three biological replicates. Correlation analyses between pairs of independent biological replicates were performed (Pearson’s correlation coefficient).

### miR-71 knockdown

In order to search for the best knockdown strategy for miR-71 in primary cell culture of *E*. *multilocularis* we evaluated 3 chemically modified anti-miRs (Fig 1), all containing substitutions of one of the no-bridging phosphate oxygen atoms with a sulphur atom (phosphorthioate): (1) 2’-O-methyl oligonucleotides in the complete sequence (anti-miR-71-2´OMe), (2) locked nucleic acid (anti-miR-71-LNA) and (3) 2´-O-methyl oligonucleotides modification in some nucleotides (anti-miR-71-partial-2´OMe). As controls, we used scrambled oligonucleotides for the anti-miR-71-2´OMe and the anti-miR-71-partial-2´OMe conditions and a negative control oligonucleotide designed by EXIQON for the anti-miR-71-LNA. The anti-miR sequences and their corresponding scrambled and negative control are shown in S1 Table. Also, primary cell culture was electroporated without any oligonucleotide (mock control). In the primary cell cultivation system, cell aggregates that give rise to metacestode vesicles are formed from parasite stem cells within 2–3 weeks [7]. For the electroporation procedure, anti-miR was added to a final concentration of 3 μM to 100 μl of primary cell aggregate suspension in selfmade siPORT electroporation buffer [27]. Electroporation procedure was used previously in *E*. *multilocularis* primary cell culture [27]. Samples were placed into a 1 mm gap electroporation cuvette (Electroporation Cuvettes Plus-BXT) and pulsed once (200 V, 13Ω and 50 μF, time constant ∼0.6 msec). After a 12-min incubation step at 37ºC, the electroporated aggregates were washed with cDMEM-A6/B4 and carefully transferred into appropriate culture wells with cDMEM-A6/B4. The cells were cultured for 24 h prior to RNA extraction or for 30 days by observation every 2 days under an inverted light microscope (Leica DM IRB). Vitality, phenotype and normal development of the aggregates was evaluated comparing the cells treated with each type of anti-miR to those treated with scrambled or negative controls, with mock electroporated aggregates and with aggregates without electroporation. This last control was used to check the normal development of the metacestodes vesicles used in the experiment.

### Total RNA isolation after knockdown experiments

Total RNA was extracted from *E*. *multilocularis* primary cell samples after 24 h in culture (Fig 1) using Direct-zol^TM^ RNA MiniPrep (ZYMO RESEARCH) according to the manufacturer's protocol. To maximize precipitation of small RNA, the eluted RNA was incubated overnight at -20°C with the addition of 0.1 volumes of 3 M sodium acetate (pH 5.2), 2.5 volumes of ethanol and 2 μl of glycogen (10mg/ml). RNA was centrifuged at 14,000 g for 30 min at 4°C, washed in 75% ethanol, air dried at room temperature and resuspended in 20 μl of nuclease-free water. Samples were stored at -80°C until use. RNA concentration was determined using a Nanodrop 1000 spectrophotometer (Thermo Scientific) and RNA integrity was assessed with an Agilent 2100 Bioanalyzer (Agilent Technologies) using a RNA 6000 Nano chip.

### RT-qPCR analysis of miR-71 in *Echinococcus multilocularis* primary cell culture after knockdown experiments

Knockdown of miR-71 and RT-qPCR (retrotranscription followed by real time polymerase chain reaction) was performed. For this, miRNA cDNA synthesis was performed as in Macchiaroli et al. [16] using 5 ng of input RNA. PCR was performed in a StepOne Plus cycler (Applied Biosystems, U.S.A.). The PCR mix consisted of 5 x HOT FIREPol EvaGreen qPCR Mix Plus 2 μl (1x), 0.4 μl (200nM) of each primer, 5.2 μl of water DNAse free and 2 μl of diluted cDNA. The cycling conditions were: 15 min at 95°C, followed by 40 x (15 s at 95°C, 20 s at 60°C) and a final step of 30 s at 72°C. Data collection was performed at 72°C. For primer design, the mature miRNA sequences identified in this work were used (S2 Table). In order to find a reference gene for normalization of the qPCR data, those miRNAs that showed high expression in primary cells of *E*. *multilocularis* were evaluated. The data were analysed by two programs: NormFinder [28] and BestKeeper [29]. The miR-4989 was chosen as reference gene since it fulfilled the stability requirements: i) low stability index according to NormFinder analysis ii) Cp standard deviation ≤0,5 with coefficient of correlation ~1 according to BestKeeper analysis (S1 Fig). Primer sequences, amplicon size and PCR efficiency values for all the primers are described in S2 Table.

### Source of genomic and annotation data

The genomic, annotation and CDS transcript data of *E*. *multilocularis* [30] are based on the EMULTI002 assembly deposited in the NCBI (National Center for Biotechnology Information) database and was downloaded from the WormBase Parasite database WBPS11-WS265 (http://parasite.wormbase.org/).

### Library construction, sequencing and bioinformatics analyses of RNA-seq data after knockdown experiments

To determine the effects of miR-71 knock-down on protein coding genes, mRNA libraries (paired-end reads) were prepared using TruSeq Stranded mRNA library prep (Illumina), according to the manufacturer´s instructions except that the provided PCR mastermix was substituted for Kapa HiFi PCR mastermix. In total, 18 libraries were prepared from three biological replicates of primary cell culture of *E*. *multilocularis* (Ingrid isolate) treated with anti-miR-71-2´OMe or anti-miR-71-LNA, their respective controls (scrambled or negative control), mock and cells without electroporation. Samples treated with anti-miR-71-partial-2´OMe were not sequenced (see methods). Samples were sequenced with a read length of 75bp and library depth of 2–3 Gb at the Wellcome Trust Sanger Institute, United Kingdom using an Illumina HiSeq v4 sequencer. The RNA-seq data are available in European Nucleotide Archive (ENA) accession number ERP 106379.

The quality of the Illumina raw sequences produced by deep sequencing and the sequences produced after removing the adaptors, were analysed with FastQC and MultiQC (http://www.bioinformatics.babraham.ac.uk/projects/fastqc). To remove the adaptors, Trimmomatic tool [31] was used and the parameters used were as follows: LEADING:3 TRAILING:3 SLIDINGWINDOW:4:15 MINLEN:45. The processed reads were first mapped to the *E*. *multilocularis* genome with HISAT2 [32] with the options—fr—dta. The assemble transcript tool StringTie [32] was used for assembling the reads in transcripts to explain the data and in this case the parameters were: -G -A -C -B -e -M 0.45. A file containing the reference gene models of *E*. *multilocularis* was used to guide the genome alignment and the StringTie assembly. This network flow algorithm can reconstruct transcripts more accurately than some previous methods [32,33] because it computes abundance and exon–intron structure at the same time. For differential expression analysis, a gene read-count data matrix was produced with the script prepDE.py provided by StringTie authors.

The DESeq2 Bioconductor package [34], a gene expression analysis tool based on the negative binomial distribution, was used for the exploratory data analysis of the gene-count matrix of the anti-miR-treated and control samples. An initial pre-filtering of the expression matrix was used to remove those genes with less than 10 reads in total, keeping 9,703 genes for the following steps of size factors and dispersion estimations. Normalized and variance-stabilized (using option “blind = TRUE” within DESeq2 Bioconductor package) transformed data was used for a principal component analysis of the top 500 highest-transcribed genes. This analysis shows the samples in the 2D plane spanned by the first two principal components, allowing exploring the effect of experimental covariates, batch effects and helping to visualize sample-to-sample distances. For differential expression, a single factor analysis was performed per anti-miR library, with log2 fold changes and Wald test *p* values calculated for the comparison anti-miR versus the suitable reference control level. *P* values were adjusted by FDR correction and shrinkage of log2 fold change estimations were performed following a normal prior distribution [34]. The code and data for differential expression analysis are available at https://github.com/natinreg/PNTD_miR-71.

### Bioinformatic prediction of miR-71 target genes in *Echinococcus multilocularis*

To identify potential targets of miR-71 in *E*. *multilocularis*, three hundred nucleotides were extracted downstream from the stop codon of each *E*. *multilocularis* coding gene using customs scripts as previously performed for *T*. *solium* [19]. The miRanda algorithm [35] was used to perform the prediction of miR-71 target sites using the predicted set of *E*. *multilocularis* 3′UTRs. The parameters used were: i) strict seed pairing; ii) score threshold: 140; iii) energy threshold: -17 kcal/mol, iv) gap open penalty: -9; v) gap extend penalty: -4; vi) scaling parameter: 4.

### RT-qPCR of selected miR-71 target genes of *Echinococcus multilocularis* primary cell culture

To validate the differential expression of three selected potential targets of miR-71: serine:threonine protein kinase (EmuJ_000250800), T cell immunomodulatory protein (EmuJ_000440000) and frizzled (EmuJ_000085700) between treated cells with the anti-miR-71-2´OMe and scrambled-2´OMe, RT-qPCR for the same three biological replicates analysed in the RNA-seq was performed (see methods). For this, cDNA synthesis was performed as previously described [5], using 80 ng of total RNA as input. Quantitative PCR (qPCR) was performed in a StepOne Plus cycler (Applied Biosystems, U.S.A.). The PCR mix consisted of 5 x HOT FIREPol EvaGreen qPCR Mix plus 2.4 μl (1x), 0.72 μl (300nM) of each primer, 6.96 μl of DNAse free water and cDNA. The cycling conditions were: 15 min at 95°C, followed by 40 x (15 s at 95°C, 20 s at 60°C) and a final step of 20 s at 72°C. Data collection was performed at 72°C. For normalization, RT-qPCR with the constitutive gene *em-elp* (EmuJ_000485800) was performed [36]. Primer sequences, amplicon size and PCR efficiency values for the primers are described in S3 Table.

## Results

### miR-71 is the highest expressed miRNA in *Echinococcus multilocularis* primary cell cultures

**Fig 2:**
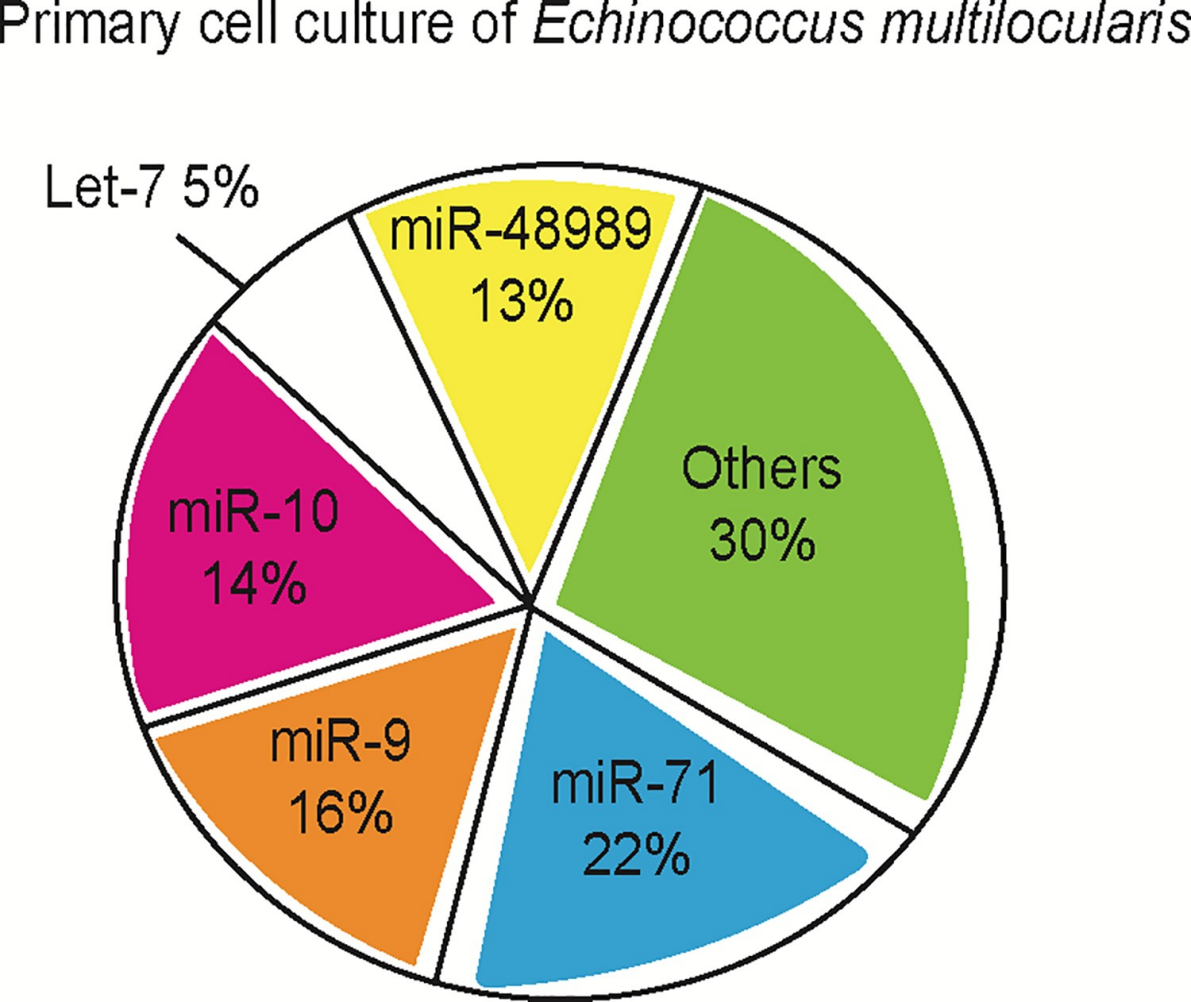
Top five most abundant miRNAs in *Echinococcus multilocularis* primary cell culture at 48 h. The average proportion of top five most abundant miRNA reads relative to the total number of mature miRNAs is shown.

We previously showed that the *E*. *multilocularis* miRNA repertoire comprises 39 mature members, all of which are expressed in the metacestode stage [15]. In the present work we determined the miRNA profile in primary cell cultures, freshly established from metacestode vesicles (S1 Table). Correlation analyses between pairs of biological replicates indicated high technical reproducibility and low biological variation (r > 0.95). We found that several miRNAs were highly expressed in primary cell culture. In particular, miR-71, miR-9, miR-10, let-7 and miR-4989 (a member of miR-277 family) represented the top five most abundant miRNAs and accounted for about 70% of total miRNA expression (Fig 2). miR-71 displayed the highest expression level in *E*. *multilocularis* primary cell cultures.

### miR-71 is critical for normal development of *Echinococcus multilocularis in vitro*

**Fig 3:**
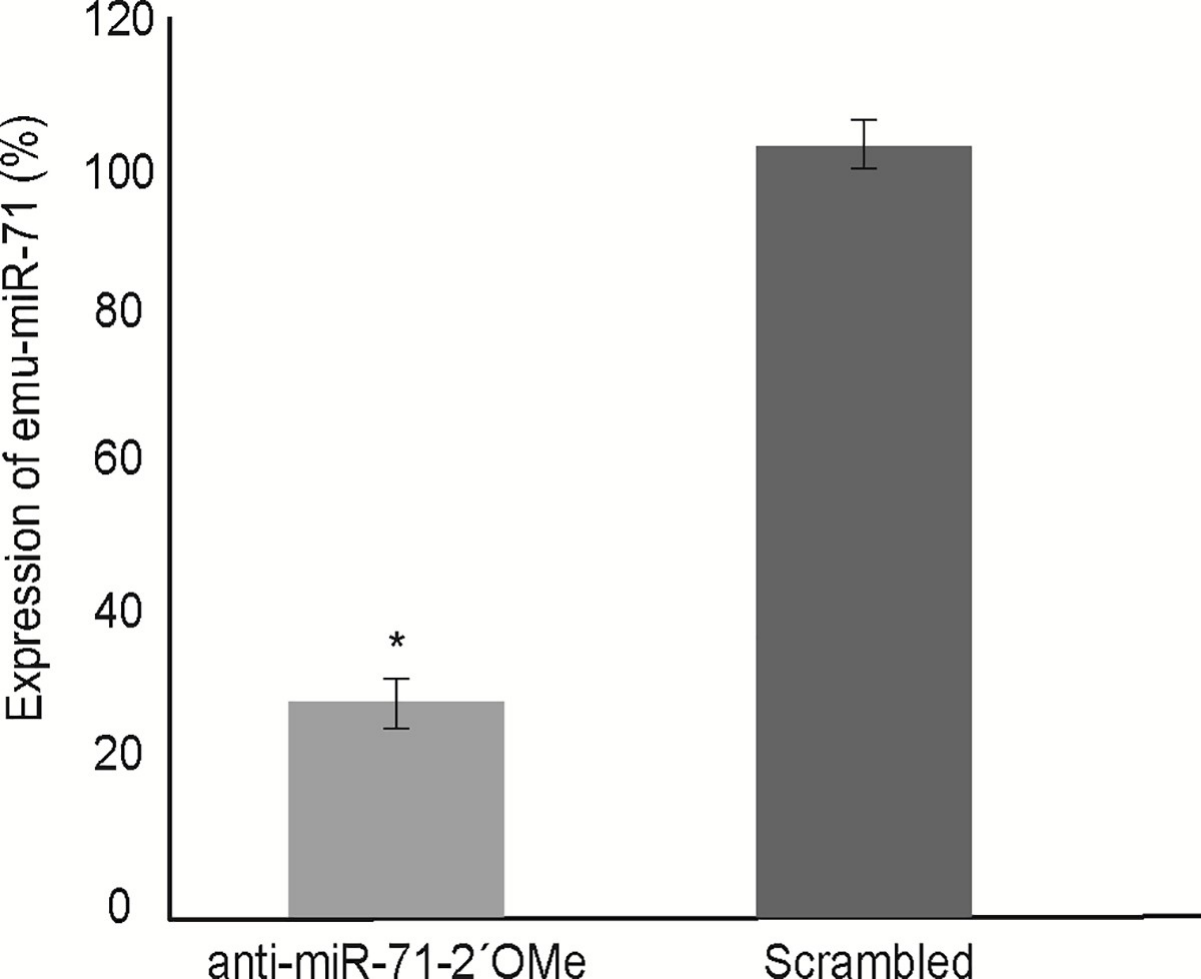
Knockdown of *Echinococcus multilocularis* miR-71 in primary cell culture with 2’-O-methyl oligonucleotides (anti-miR-71-2´OMe). The primary cell culture was electroporated with anti-miR and the respective scrambled control. The effects on the levels of endogenous miR-71 was determined by RT-qPCR at 24 h post-electroporation (48 h culture). Data illustrate representative results with the mean and standard error derived from three technical replicates for each of the three independent biological replicates. The results were calibrated with the average (normalized ct) of the cells treated with the scrambled oligonucleotide. * P ≤ 0.05.

**Fig 4:**
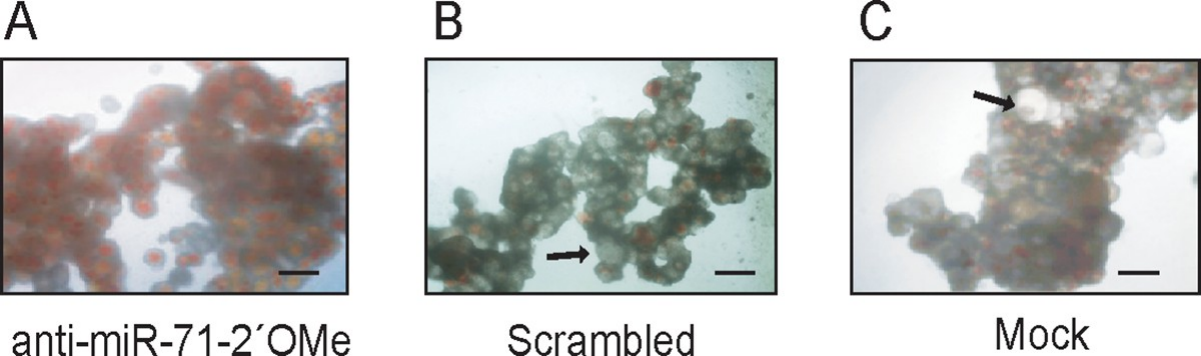
Light microscope images of *Echinococcus multilocularis* primary cell culture 4 weeks post electroporation. (A) Cells transfected with anti-miR-71-2’OMe by electroporation. The red cavities have been maintained and metacestodes vesicles were not observed. (B) Cells transfected by electroporation with scrambled-2´OMe control in which young cysts (arrow) developed. (C) Mock electroporated cells in which young cysts (arrow) developed. Bars indicate 50 μm.

RNA knockdown approaches against mRNAs have previously been carried out in *E*. *multilocularis* [27] and were strongly dependent on electroporation of primary cells. In this work, we evaluated the best strategy for miR-71 knockdown in primary cell culture of *E*. *multilocularis* using three chemically modified anti-miRs (Fig 1). At 24 h post transfection (48 h cell culture), cells treated with anti-miR-71-2’OMe showed a 78.4% (± 3.4) reduction of miR-71 expression (Fig 3). Likewise, cells treated with anti-miR-71-LNA and anti-miR-71-partial-2´OMe showed a reduction of miR-71 expression (S2 Fig). After 30 days of cultivation, clear phenotypic alterations were observed (Fig 1). In control and mock cultures, parasite cells developed as previously described [7] and first formed cellular aggregates with small, red cavities (S3A Fig) which probably result from an accumulation of phenol red provided by the culture medium. After around one week of incubation, the cellular aggregates enlarged due to germinative cell proliferation [5,7] and most of the cavities lost red staining, most probably due to the formation of a tegument that excludes phenol red. At the end of development, after around 2–3 weeks, mature metacestode vesicles were formed in culture (S3B–S3D Fig). On the other hand, the development in cell cultures transfected with anti-miR-71-2´OMe was blocked at the first stage and displayed clear morphological differences when compared to scrambled and mock controls (Fig 4). Cultures transfected with anti-miR-71-2´OMe consistently contained red cavities which did not increase in size and remained stained throughout incubation. Also, we did not observe the formation of mature metacestode vesicles in these cultures, at least until three weeks of incubation. In transfections performed with the other chemically modified anti-miRs, no change at the phenotype level was observed with respect to controls (S4 Fig). Taking into account that partial 2´OMe was the less effective modified oligonucleotide in miR-71 knockdown and that phenotypic effects were not observed we did not continue the analysis with this modified anti-miR.

### miR-71 regulates relevant targets at the beginning of the development of *Echinococcus multilocularis* metacestodes *in vitro*

According to the canonical role of miRNAs in gene regulation [14], we expected an up-regulation of miR-71 target gene expression upon miR-71 knockdown. We thus explored by RNA-Seq the expression profiles of predicted miR-71 targets. To this end, RNA was analyzed from controls and from miR-71 knockdown samples 24 h after electroporation (Fig 1). We obtained between 11 and 15 million high quality paired-end reads per sample, with 98–99% overall mapping rate to the *E*. *multilocularis* genome (S5 Table).

**Fig 5:**
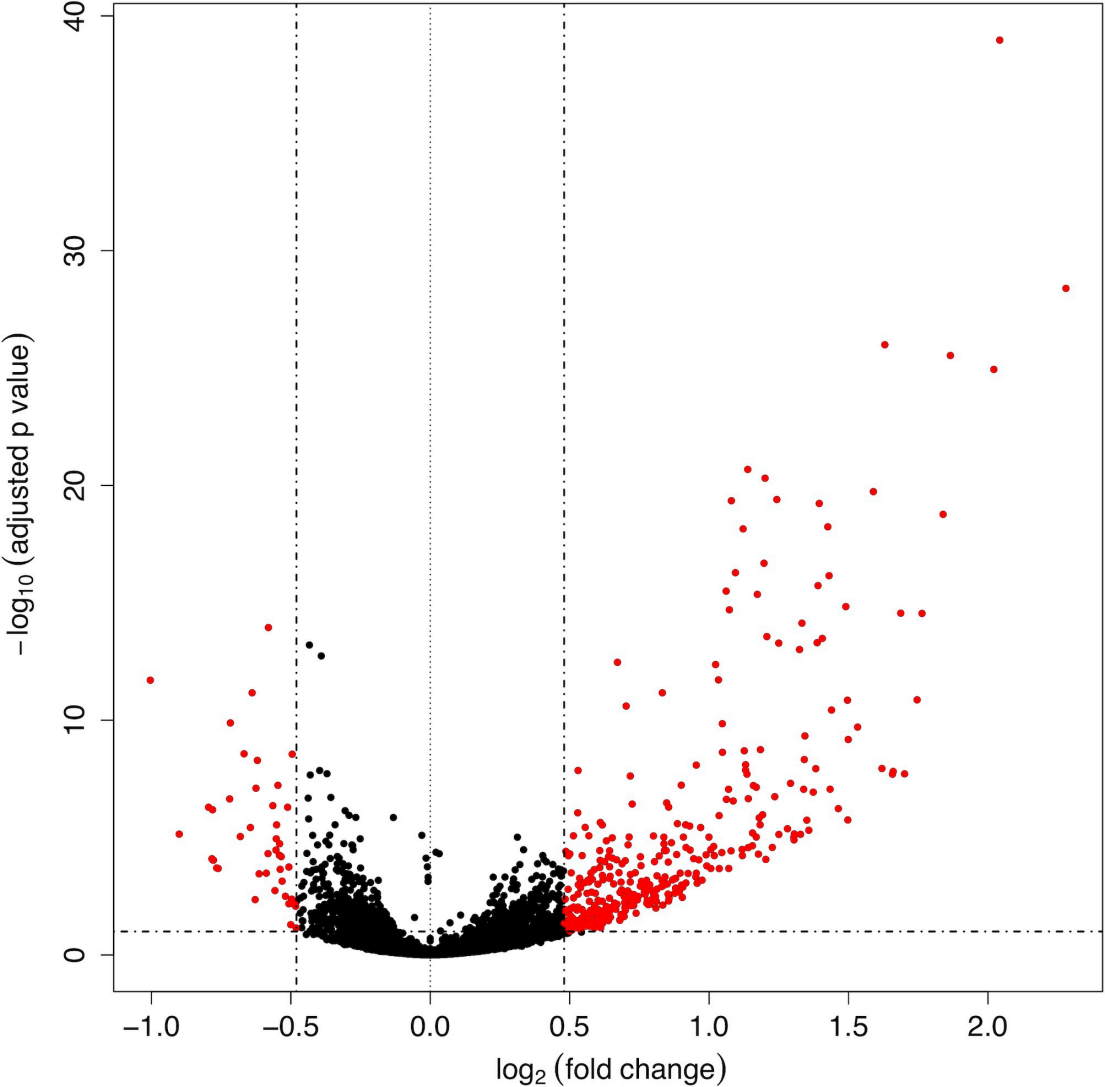
Volcano plot shows the gene statistical significance (on the y-axis) over the log2 fold change after normal shrinkage (on the x-axis) for all the samples from differential expression analysis between anti-miR-71-2´OMe vs scramble-2´OMe control. Red points correspond to 416 differentially expressed transcripts (372 up-regulated, in the right of the plot) with an adjusted p-value less than 0.1 and an absolute log2 fold change above 0.48.

After DESeq2 normalization and variance stabilizing transformation of the gene-count expression matrix, a principal component analysis of all samples was performed (S5 Fig). Firstly, these data indicated a clear effect of electroporation on gene expression since the first component splits the not electroporated samples from all the remaining conditions. Second, the treatment with anti-miR-71-2´OMe was the only knockdown procedure showing a consistent separation of anti-miR-71 and scrambled treated samples as can be seen along the second component (anti-miR-71-2´OMe and scrambled-2´OMe sample). Our results showed that 1,292 genes significantly changed transcription levels (adjusted p-value < 0.1) upon miR-71 knockdown (S6 Table). Of these genes, 744 (57.6%) were significantly up-regulated (log2FC_shrinkage>0, adjusted p-value < 0.1) and 372 of them displayed increased expression of more than 40%. (Fig 5, red points in the right of the volcano plot).

Interestingly, although miR-71 expression was apparently downregulated upon anti-miR-71 LNA electroporation (see above) when compared to control LNA, we did not see differential gene expression between these samples (S6 Fig) in accordance with the lack of differential phenotype (S4 Fig).

**Fig 6:**
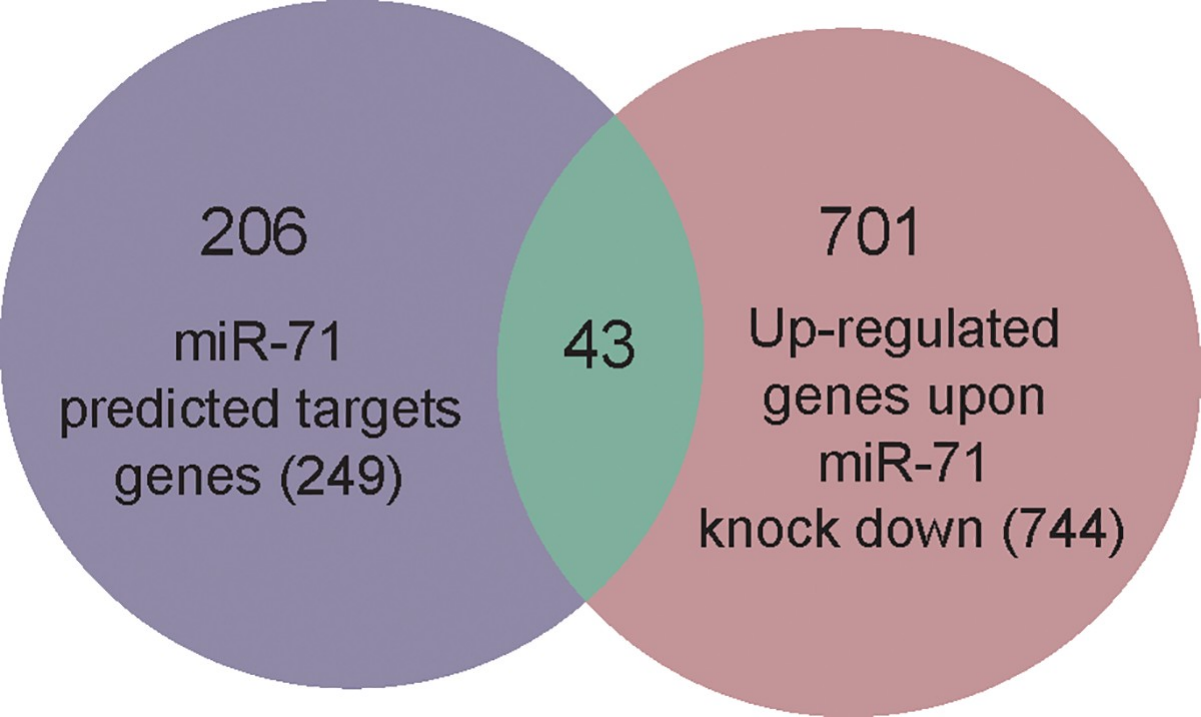
Venn diagram showing the integration of the target genes of *Echinococcus multilocularis* predicted by miRanda using genomic information, and genes over-expressed in miR-71 knocked down primary cells (P-value adjustment < 0.1).

**Fig 7:**
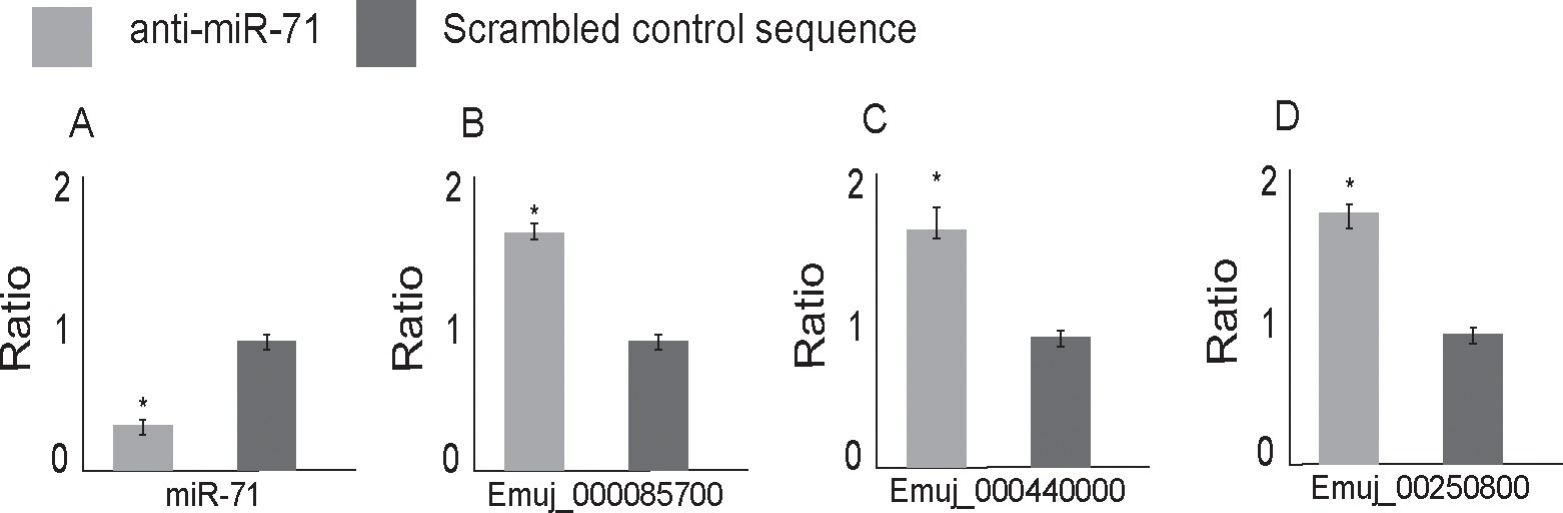
Effect of miR-71 suppression in miR-71 level (A), and in the mRNA level of the potential target genes EmuJ_000085700 (frizzled) (B), EmuJ_000440000 (T cell immunomodulatory protein) (C) and EmuJ_000250800 (serine:threonine protein kinase) (D). Primary cells were obtained from *Echinococcus multilocularis* metacestodes (Ingrid isolate). The cells were electroporated with anti-miR-71-2´OMe or scrambled-2´OMe and their effects on the levels of endogenous miRNA (A) and predicted target mRNAs (B,C,D) determined by RT-qPCR at 24 h post-electroporation (48 h cell culture). Data illustrate representative results with the mean and standard error derived from triplicate experiments. * mean P ≤ 0.05.

To identify potential miR-71 target genes, we matched it's seed sequence to 3'UTRs of *E*. *multilocularis* transcripts and predicted overall 252 candidate target sites (249 genes) (S7 Table). The up-regulated mRNAs and the predicted targets were integrated (Fig 1). After combining the list of up-regulated mRNAs (see above) and the list of candidate target genes, 43 mRNAs fulfilling both criteria were identified (Fig 6 and S8 Table). Finally, we selected from this intersection those genes with shrinkage_FC greater than 1.4 (log2 FoldChange_Shrinkage >0.48) and p adj< 0.05 (16 genes). These genes are represented in Table 1, where 17 target sites are depicted since one of the genes has two miR-71 target sites. Since evolutionary conservation of target sites provides more reliability to target predictions [37] we determined the presence of miR-71 recognition sites in the orthologous 3’UTRs from *Echinococcus canadensis*, *E*. *granulosus* and *T*. *solium* for this group of genes. We observed that 13 of the 16 selected genes showed miR-71 conserved target sites in *E*. *granulosus*, *E*. *canadensis* and/or *T*. *solium* orthologs (Table 1), adding confidence to these results. We also determined the energy of miRNA-target interaction [35], target site localization in the 3’UTR [38] and type site since these features are related to target site efficacy [14]. Eight out of the 17 target sites (47%), were located near the stop codon (at least 15 nt from the stop codon) and can thus be considered effective sites [14] (Table 1). The type of regulatory site was also analysed. We observed that all the target sites were canonical sites and belonged to the three site types with higher efficacy according to Bartel [14]: 7mer-A1, 7mer-m8 and 8mer (Table 1 and S9 Table). Among the 16 selected targets (Table 1), we found genes involved in development such as a member of the frizzled-family of GPCRs (EmuJ_000085700) for Wnt ligands that participate in canonical and non-canonical signalling [39,40]; genes related to the establishment of multicellularity such as protocadherin (EmuJ_000848200) [41]; transcription factors (EmuJ_000191100, EmuJ_001186600) [42]; serine:threonine protein kinase encoding genes that could participate in multiple cellular processes (EmuJ_000250800) [43] and genes involved in mRNA stability such as PAB dependent poly(A) specific ribonuclease [44], a protein involved in miRNA function (EmuJ_000610700) [45]. Another interesting target that participates in parasite-host communication is the T-cell immunomodulatory protein (EmTIP) (EmuJ_000440000) [12]. Additionally, three of the miR-71 targets code for proteins without annotation that are tapeworm specific: EmuJ_000085500 (also targeted by miR-281-3p) [18], EmuJ_000865800 and EmuJ_001140200. EmuJ_000865800 codes for a protein with at least one transmembrane domain and the EmuJ_000085500 encoded protein has a coiled-coil and RecF/RecN/SMC N terminal domain (S10 Table). We investigated by RT-qPCR three potential targets that were selected among the 16 genes due to their predicted role in development or host-parasite communication and found indeed increased expression of all three mRNAs, thus verifying the RNA-Seq data (Fig 7).

**Table 1:** Sixteen potential target genes of miR-71 in *Echinocuccus multilocularis* primary cell culture up-regulated after transfection with anti-miR-71-2´OMe. (log2FoldChange_Shrinkage > 0.48 and P-value adjustment < 0.05). Position of miR-71 target site in the 3’ UTR of each target gene, conservation of miR-71 targeting in *Echinococcus canadensis*, *Echinococcus granulosus* and *Taenia solium* orthologs and type of site according to Bartel [14] are also shown.

Gene ID	Annotation	FoldChange	UTR_start	UTR_end	Conservation	Type of site
EmuJ_000085500	Expressed conserved protein	1.4	14	33	*T*. *solium*, *E*. *canadensis and E*. *granulosus*	7mer-A1
EmuJ_000085700	Frizzled	1.7	156	180	*T*. *solium and E*. *granulosus*	7mer-m8
EmuJ_000124300	CTR1, high affinity copper uptake protein 1	1.5	27	50	*T*. *solium*, *E*. *canadensis and E*. *granulosus*	8mer
EmuJ_000145900	Glutamate receptor interacting protein 1	1.6	5	26	*T*. *solium*	8mer
EmuJ_000154000	E3 ubiquitin protein ligase MARCH8	1.5	33	54	*T*. *solium*, *E*. *canadensis and E*. *granulosus*	7mer-m8
EmuJ_000165500	USP6 N terminal protein	1.4	23	45	*T*. *solium*, *E*. *canadensis and E*. *granulosus*	8mer
EmuJ_000191100	Transcription factor SOX 14	1.6	54	74	*T*. *solium*, *E*. *canadensis and E*. *granulosus*	8mer
EmuJ_000228700	Leucine rich repeat, typical subtype	1.6	186	206	*T*. *solium*, *E*. *canadensis and E*. *granulosus*	7mer-m8
EmuJ_000250800	Serine:threonine protein kinase	1.4	218	239	*E*. *granulosus*	7mer-m8
EmuJ_000440000	T cell immunomodulatory protein	1.5	26	47	*T*. *solium*, *E*. *canadensis and E*. *granulosus*	8mer
EmuJ_000440000	T cell immunomodulatory protein	1.5	84	106	*E*. *canadensis* and *E*. *granulosus*	7mer-m8
EmuJ_000473600	Sodium dependent phosphate transport protein 1	1.4	154	174	*T*. *solium and E*. *granulosus*	7mer-m8
EmuJ_000610700	PAB dependent poly(A) specific ribonuclease	1.5	25	49	*n/c* ^2^	8mer
EmuJ_000848200	Protocadherin alpha 7	1.7	9	32	*n/c*	7mer-m8
EmuJ_000865800	n/a^1^	2.1	12	33	*n/c*	8mer
EmuJ_001140200	Expressed conserved protein	2.8	169	191	*n/c*	8mer
EmuJ_001186600	Ets variant gene 4	1.6	25	46	*T*. *solium*	7mer-m8

^1^ Without annotation in Wormbase parasite

^2^ Without conservation in *T*. *solium*, *E*. *canadensis* and *E*. *granulosus*

## Discussion

In this study, the role of miRNAs in the early development of *E*. *multilocularis* was addressed by performing miRNA knockdown experiments. To our knowledge, this is the first report of successful miRNA knockdown in tapeworms. miR-71 was selected for knockdown experiments since, among other features, miR-71 displayed high expression levels in *E*. *multilocularis* primary cells as well as in the larval stages of several cestodes [19]. High abundance of an individual miRNA is considered functionally important since several miRNA complementary sequences usually compete for actual miRNA target sites [14]. The results of this work suggest that *E*. *multilocularis* miR-71 is necessary for proper early development of metacestode vesicles *in vitro*.

Since there were no previous reports on successful miRNA knockdown in cestodes, we herein assayed different types of chemically modified anti-miRs to optimize protocols for effective functional genomics in these organisms. We found that transfection by electroporation with 2’-O-methyl fully modified oligonucleotide antisense to *E*. *multilocularis* miR-71, significantly reduced miR-71 levels. This is probably due to miR-71 degradation since it is known that this type of modified oligonucleotides promote miRNA degradation [46,47]. Notably, cells transfected with this oligonucleotide showed a different phenotype from control oligonucleotide-transfected cells and did not develop metacestode vesicles. Since primary cell culture is enriched in stem cells which are able to produce metacestode vesicles [5], this result suggests that miR-71 regulates early events that are important for parasite development, probably involving stem cells. Furthermore, significant changes in mRNA levels were observed in ~16% of protein coding genes, suggesting that miR-71 knockdown altered the primary cell culture transcriptome. It is interesting to note that antisense oligonucleotides with 2'OMe modification were also already used to successfully alter miRNA expression in *S*. *japonicum* [22], suggesting that this type of modification is important for establishing functional genomics methodology in flatworms. The level of target mRNA upregulation observed upon miRNA knockdown was similar to our results, since we observed that most miR-71 mRNA targets were up-regulated between 1.4 and 1.6 times. This modest level of repression is typical for miRNA regulation and has also already been described in other studies [14]. We also attempted knockdown of miR-71 using LNA anti-miR but neither phenotypic alterations nor changes in mRNA levels were observed although miR-71 levels were decreased as measured by RT-qPCR. One possible explanation for these results is that most LNA molecules were retained in endosomes [48] and were not able to interact with cytosolic miR-71. The LNA anti-miR retained in endosomes could have been released after cell lysis to form strong heteroduplex with the miRNA [49], which is characteristic of this type of oligonucleotides and often causes false results in RT-qPCR.

The combined approach employed in this work allowed identifying possible miR-71 target genes. Importantly, the transcriptomic studies performed allowed to determine that miR-71 and the putative target genes are co-expressed in *E*. *multilocularis* primary cell culture, which is a necessary condition for target validation. Among these genes, some are presumably involved in parasite development such as a frizzled-family member of GPCRs which presumably acts in the Wnt signaling pathway. This evolutionarily conserved pathway regulates crucial aspects of cell fate determination, cell migration, cell polarity, neural patterning and organogenesis in metazoan embryonic development [50]. *E*. *multilocularis* and other flatworms possess genes associated with each of the three known Wnt pathways [51,52], and recently Koziol et al. [53] showed that the Wnt pathway is involved in anterior-posterior specification in *E*. *multilocularis* larvae. In concordance with our results, this signalling pathway has recently also been shown to be regulated by miRNAs in the platyhleminth *S*. *japonicum* [22]. We also found an ortholog of Serine:threonine protein kinases among the *Echinococcus* miR-71 targets. Protein kinase function is evolutionarily conserved from *Escherichia coli* to human and plays a role in a multitude of cellular processes, including division, proliferation, apoptosis, and differentiation [43]. Another miR-71 possible target, bearing 2 recognition sites in its 3’ UTR, is EmTIP. This gene is an ortholog of the human T-cell immunomodulatory protein, TIP [54]. The putative target site for miR-71 in this gene is conserved in *E*. *canadensis* and *T*. *solium*. This protein was shown to be necessary for *E*. *multilocularis* primary stem cell proliferation and metacestode development and it was suggested that it could be associated with cell-cell/extracellular matrix interactions [12]. Also, it stimulates IFN ɣ secretion from human CD4+ T-cells, suggesting that this protein could be important for host-parasite communication. Our results suggest that, by controlling EmTIP expression, miR-71 could be involved in the regulation of establishment of multicellularity and regulation of host immune response. Other interesting possible miR-71 targets are protocadherin, a cell adhesion protein linked with multicellularity establishment [41] and two transcription factors orthologs that play important roles in various biological processes [55]. Interestingly, miR-71 could also target PAB dependent poly(A) specific ribonuclease, a deadenylation protein required for miRNA-mediated silencing in several model organisms [45]. Thus, miR-71 seems to downregulate the expression of a protein necessary for miRNA function, suggesting that it can constitute a negative feedback loop.

Bioinformatically predicted miRNA targets that did not show upregulation upon miRNA knockdown can be false-positive. However, we cannot discard all of them as miR-71 targets since under the conditions assayed, the miRNA recognition site could be bound to other (regulatory) molecules and/or the 3’UTR could adopt a structure that does not allow miRNA binding. In other conditions or life cycle stages these genes might nevertheless be targeted by miR-71. On the other hand, variation in expression levels of genes that were not predicted miR-71 targets can be explained as indirect effects of miR-71 knockdown. Another possibility is that some of these genes are real targets but were not predicted as a consequence of the stringency of the parameters used for target prediction.

Due to their involvement in gene regulation, miRNAs have been identified as high-value targets for therapy. Several miRNA-targeting drugs are now in clinical trials or even close to market launch [56], including miRNA-targeting drugs against viral pathogens [57]. Inhibition of an endogenous miRNA can be achieved by the delivery of antisense oligonucleotides that bind and inhibit its interaction with the mRNA target. This approach has challenges such as the delivery into the cell of oligonucleotides that are poorly cell-permeable, and consequently, delivery strategies such as encapsulation into nanoliposomes are needed [58]. However, miRNA targeting has advantages over small molecule drugs directed against proteins. Since one miRNA can regulate the expression of several protein coding genes, by inhibiting one miRNA, it is theoretically possible to target several proteins. Interestingly, the use of small molecule modulators to target specific miRNAs is being addressed as an alternative strategy of miRNA-based therapy [56]. Parasitic helminth miRNAs have been proposed as potential targets of intervention strategies [59–61]. Furthermore, targeting parasite miRNAs that are protostome-specific, or bilaterian-specific but absent or divergent from host orthologs, could lead to selective drugs. Focusing on *E*. *multilocularis*, available drugs, i.e. benzimidazoles, are only parasitostatic probably due to their inability to target the key parasite stem cell population [13]. By inhibiting miR-71, as shown in this study, which is probably highly expressed in parasite stem cells, these problems could possibly be circumvented.

Taken together, the high miR-71 expression in stem-cell enriched primary culture of *E*. *multilocularis*, the inhibition of parasite early development by interfering miR-71 expression, the target genes related with parasite development and the high proportion of genes with altered expression after miRNA knockdown, suggest that this miRNA is a master regulator of gene expression in *E*. *multilocularis*. Furthermore, miR-71 is absent in the vertebrate host, suggesting that this miRNA is a potential selective drug target. The findings of the present study unveil miR-71 function in *Echinococcus* development and provide a methodology for miRNA functional analysis in this parasite that could be applied to related tapeworms.

## Supporting information

S1 Fig NormFinder and BestKeeper summarizing results. miR-4989 was the gene that kept low stability index according to NormFinder analysis and CP standard deviation ~0,5 with coefficient of correlation ~1 according to BestKeeper analysis. (A) Primary cell culture transfected with anti-miR-71-2’-O-methyl chemical modification in the complete sequence (anti-miR-71-2´OMe), (B) Primary cell culture transfected with anti-miR-71-locked nucleic (anti-miR-71-LNA).
(TIF)

S2 Fig Knockdown of *Echinococcus multilocularis* miR-71 in primary cell culture with (A) locked nucleic acid (LNA) and (B) 2’-O-methyl oligonucleotides in some nucleotides (Partial-2´OMe). The primary cell culture was electroporated with anti-miR and the respective scrambled control. Their effects on the levels of endogenous miR-71 was determined by RT-qPCR at 24 h post-electroporation. Data illustrate representative results with the mean and standard error derived from triplicate experiments. The results were calibrated with the average (ct) of the mock. * mean P ≤ 0.05
(TIF)

S3 Fig Normal development of the primary cell culture of *Echinococcus multilocularis*. (A) Cells after 10 days of cultivation. Arrow indicates the first red cavities. (B) 15–20 days later the aggregates started to create bigger cavities (arrow). (C) 20–30 days from the first day of cultivation only few rad cavities are observed. (D) Mature metacestode without red staining (arraow) are observed.
(TIFF)

S4 Fig Light microscope images of *Echinococcus multilocularis* primary cell culture 4 weeks post electroporation. Transfected cells with (A) anti-miR-71-LNA (B) Negative-control-LNA (C) anti-miR-71-partial-2´OMe (D) Scrambled-partial-2´OMe control. Transfected cells by electroporation with the anti-miRs and respective controls showed the development of metacestode vesicles (arrows) for all conditions. Bars indicate 50 μm.
(XLS)

S5 Fig Principal component plot of the samples from *Echinococcus multilocularis* primary cell culture treated with anti-miRs, with their respective controls including cell culture without electroporation. 2OME with yellow label (1,2,3): Biological replicates of primary cell culture treated with anti-miR-71-2´OMe. 2OMES with red label (1,2,3): Biological replicates of primary cell culture treated with Scrambled-2´OMe. LNA with pink label (1,2,3): Biological replicates of primary cell culture treated with anti-miR-71-LNA. LNAS with blue label (1,2,3): Biological replicates of primary cell culture treated with Negative-Control-anti-miR-71-LNA. CE with green label (1,2,3): Biological replicates of primary cell culture electroporated (Mock). CSE with light blue label (1,2,3): Biological replicates of primary cell culture without any treatment.
(TIF)

S6 Fig *MA-plot* function (*DESeqDataSet*) shows the log2 fold change attributable to a given variable over the mean of normalized counts for all the samples form differential expression between anti-miR-71-LNA vs Negative-control-LNA. No differential expression is observed (*p* value less than 0.1).
(TIF)

S1 Table anti-miR and control sequences used in the knockdown experiments of miR-71 in *Echinoccocus multilocularis* primary cell culture. (XLS)

S2 Table Sequences of mature miRNAs, primers and RT-qPCR data. (XLS)

S3 Table Primer sequences of 3 predicted target genes of miR-71 in *Echinococcus multilocularis* primary cell culture and the reference gene for the qPCR. (XLS)

S4 Table Mature, star and precursor sequences of *Echinococcus multilocularis* miRNAs identified in the primary cell culture (48h). (XLS)

S5 Table RNA-seq data for *Echinococcus multilocularis* (Ingrid isolate) primary cell culture transfected with anti-miR-71 and their respective controls after 24 hrs. Raw paired-end reads were 75 bp long. Quality and adapter trimming was performed with Trimmomatic, for a minimum final length of 45 bp. Genome alignment and transcript assembly and quantification was performed with HISAT2 plus StringTie. Expression values are given as transcripts per million (TPM).
(XLS)

S6 Table DESeq2 analysis of 9,703 expressed transcripts in *E. multilocularis* primary cells, for treatment with anti-miR-71-2´OMe (anti-miR-71-2´OMe versus scrambled-2´OMe samples). (XLS)

S7 Table Bioinformatic target prediction for miR-71 in *Echinococcus multilocularis* genome. (XLSX)

S8 Table Forty-three out of the 249 predicted targets over-expressed in anti-miR-71-2´OMe treated primary cells with respect to primary cells treated with the scramble-2´OMe. (XLS)

S9 Table Analysis of the different types of miR-71 regulatory sites for the 16 selected potential targets in *Echinococcus multilocularis*. (XLSX)

S10 Table Conservation and domain analysis for 3 miR-71 selected target genes of unknown function. (XLS)
