# Supplementary figures and images for: Deciphering the role of miR-71 in Echinococcus multilocularis early development in vitro
Source: PLoS Negl Trop Dis. 2019 Dec 27;13(12):e0007932. doi: 10.1371/journal.pntd.0007932 (PMC6957206; doi:10.1371/journal.pntd.0007932)

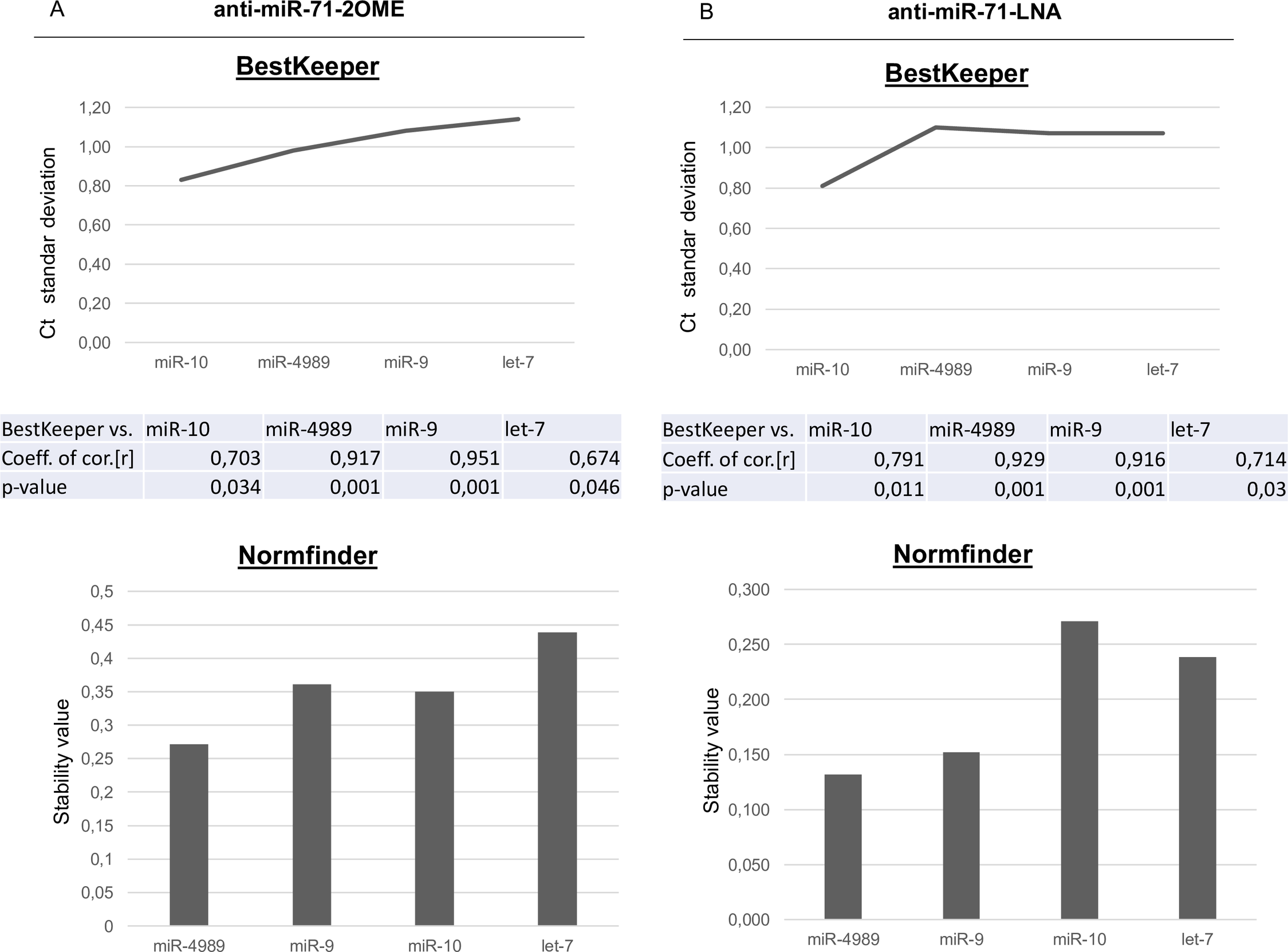

Supplement: S1 Fig — miR-4989 was the gene that kept low stability index according to NormFinder analysis and CP standard deviation ~0,5 with coefficient of correlation ~1 according to BestKeeper analysis. (A) Primary cell culture transfected with anti-miR-71-2’-O-methyl chemical modification in the complete sequence (anti-miR-71-2´OMe), (B) Primary cell culture transfected with anti-miR-71-locked nucleic (anti-miR-71-LNA). (TIF) [file pntd.0007932.s001.tif]

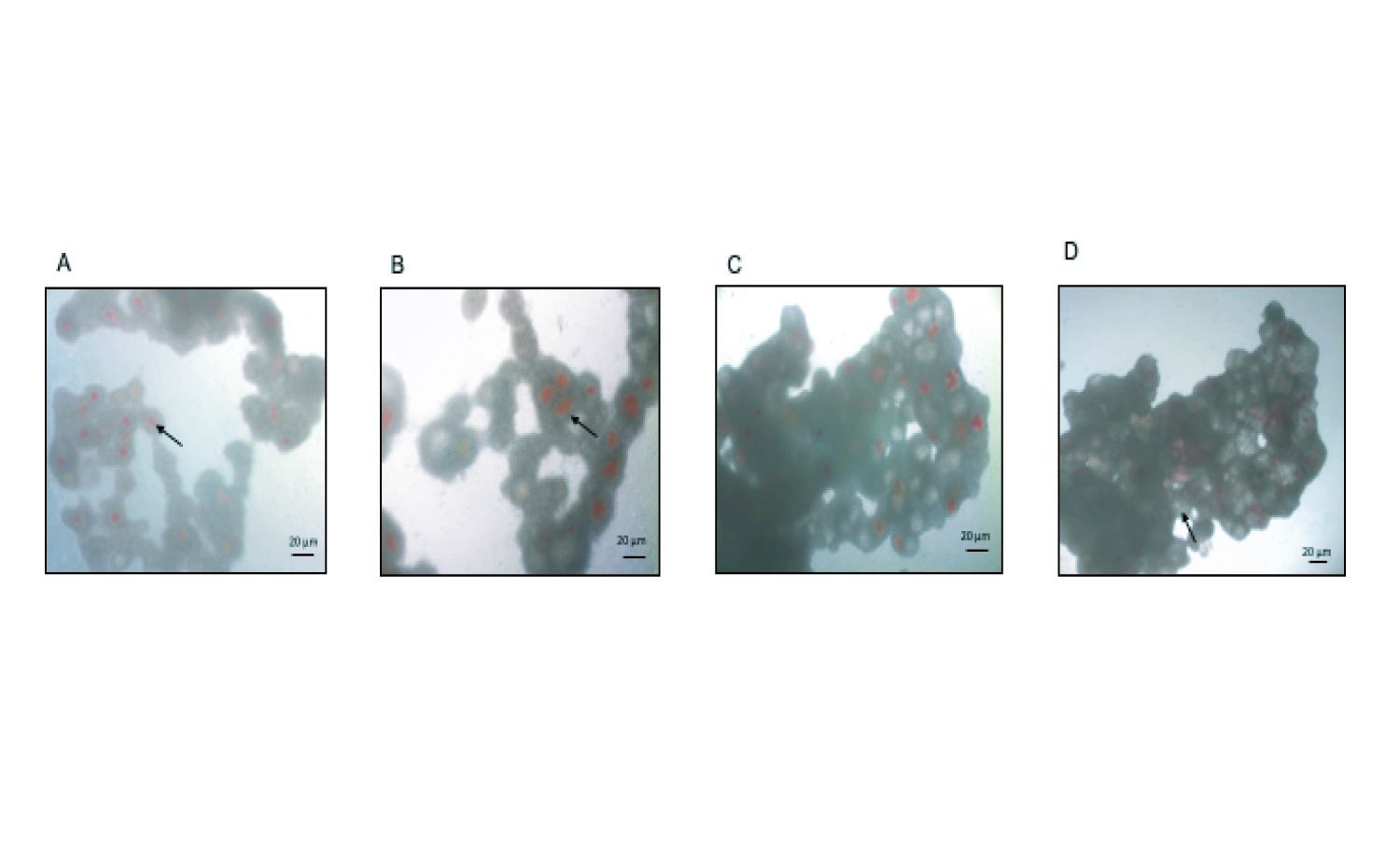

Supplement: S2 Fig — Knockdown of Echinococcus multilocularis miR-71 in primary cell culture with (A) locked nucleic acid (LNA) and (B) 2’-O-methyl oligonucleotides in some nucleotides (Partial-2´OMe). The primary cell culture was electroporated with anti-miR and the respective scrambled control. Their effects on the levels of endogenous miR-71 was determined by RT-qPCR at 24 h post-electroporation. Data illustrate representative results with the mean and standard error derived from triplicate experiments. The results were calibrated with the average (ct) of the mock. * mean P ≤ 0.05 (TIF) [file pntd.0007932.s002.tif]

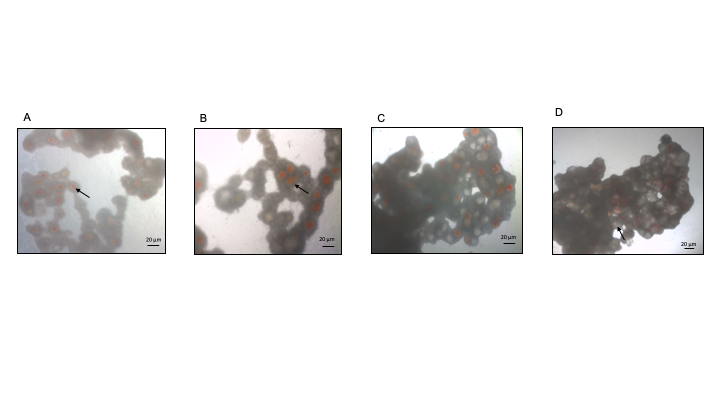

Supplement: S3 Fig — (A) Cells after 10 days of cultivation. Arrow indicates the first red cavities. (B) 15–20 days later the aggregates started to create bigger cavities (arrow). (C) 20–30 days from the first day of cultivation only few rad cavities are observed. (D) Mature metacestode without red staining (arraow) are observed. (TIFF) [file pntd.0007932.s003.tiff]

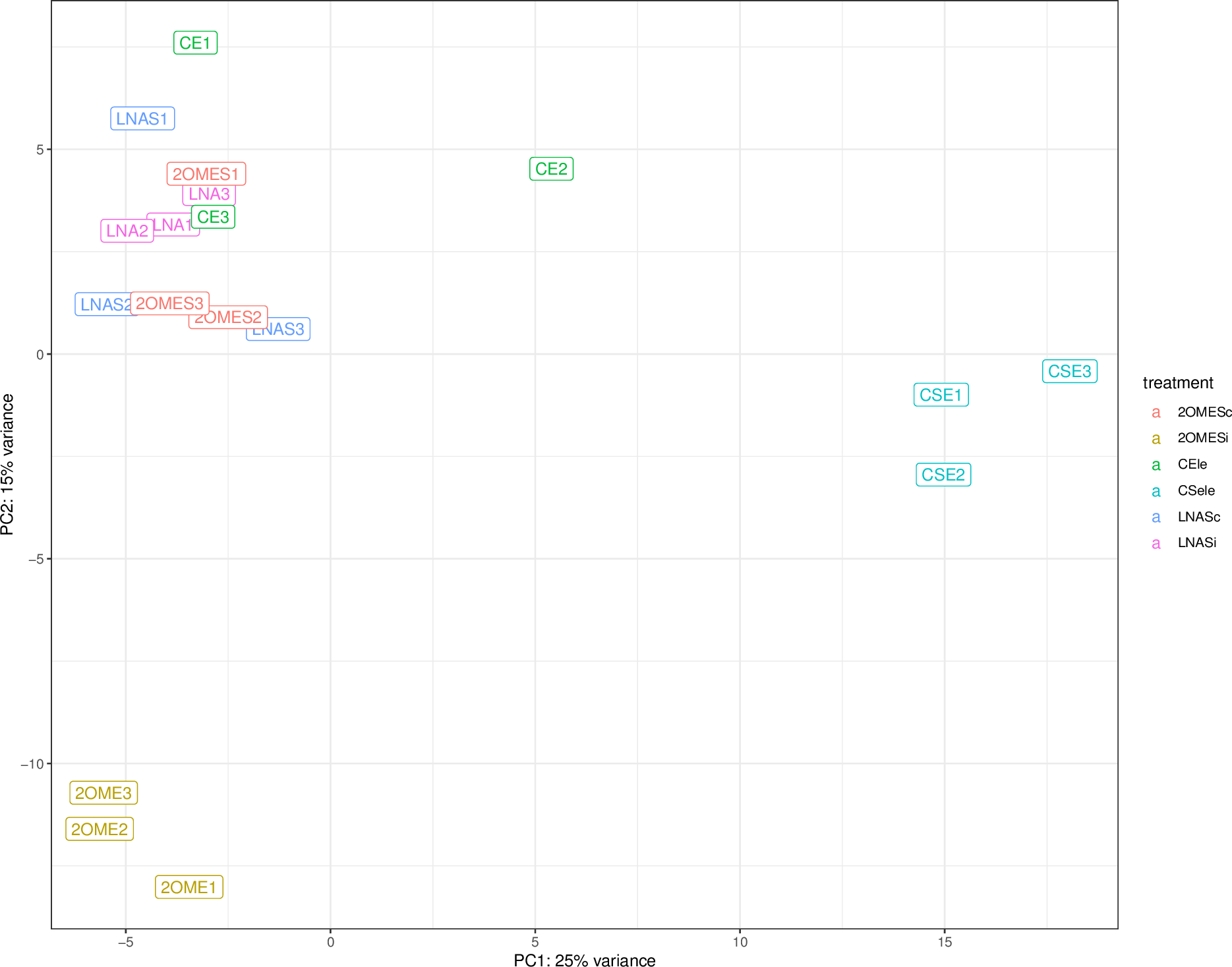

Supplement: S5 Fig — 2OME with yellow label (1,2,3): Biological replicates of primary cell culture treated with anti-miR-71-2´OMe. 2OMES with red label (1,2,3): Biological replicates of primary cell culture treated with Scrambled-2´OMe. LNA with pink label (1,2,3): Biological replicates of primary cell culture treated with anti-miR-71-LNA. LNAS with blue label (1,2,3): Biological replicates of primary cell culture treated with Negative-Control-anti-miR-71-LNA. CE with green label (1,2,3): Biological replicates of primary cell culture electroporated (Mock). CSE with light blue label (1,2,3): Biological replicates of primary cell culture without any treatment. (TIF) [file pntd.0007932.s005.tif]

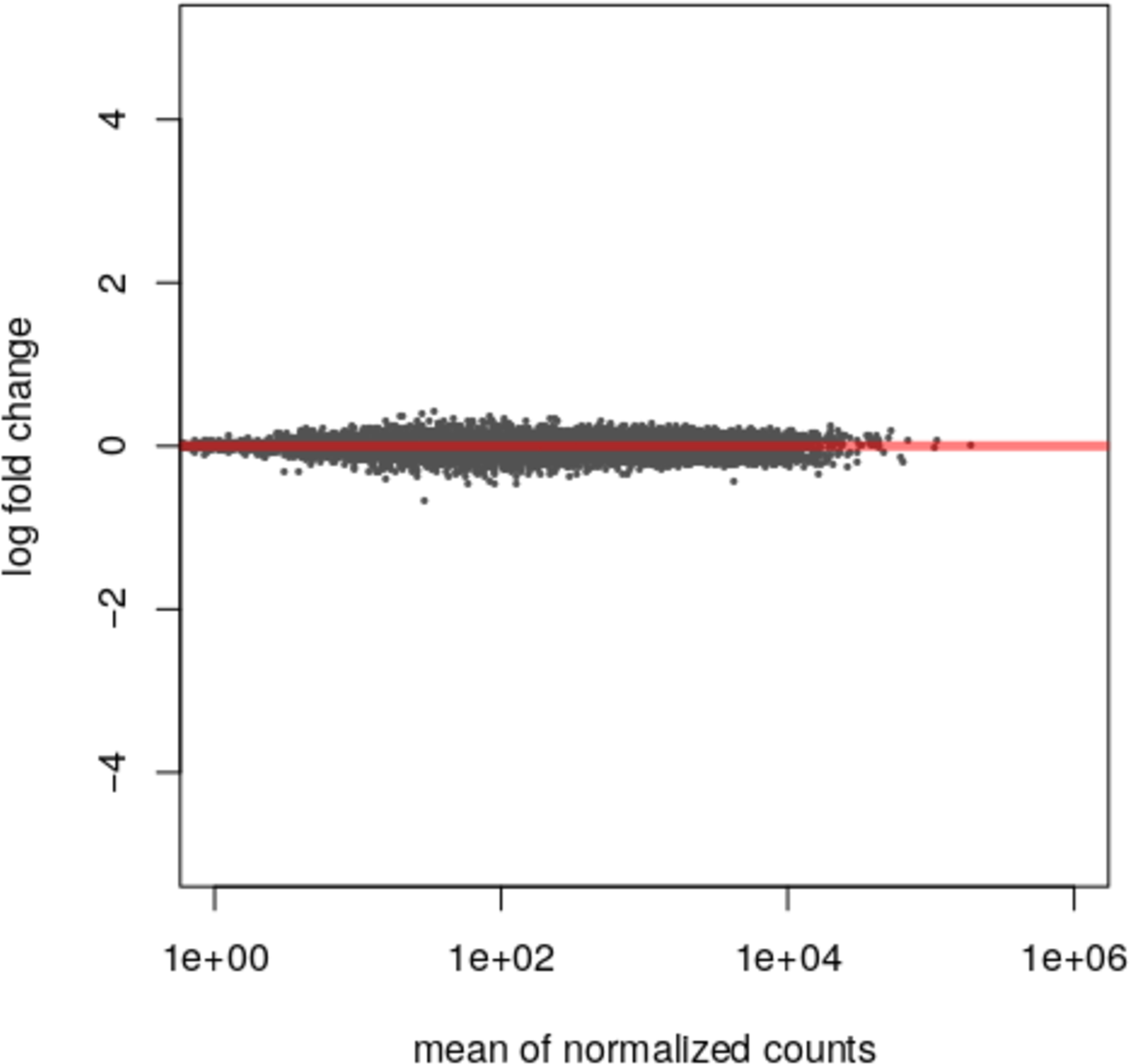

Supplement: S6 Fig — No differential expression is observed (p value less than 0.1). (TIF) [file pntd.0007932.s006.tif]
